# Diquat-induced organ toxicity: a focus on regulated cell death pathways and mitochondrial dysfunction

**DOI:** 10.3389/fcell.2026.1820625

**Published:** 2026-07-07

**Authors:** An-Bu Liu, Yue Shen, Li-Shan Yang, Lei Ma, Jun-Fei Zhang

**Affiliations:** 1 Department of Emergency Medical, General Hospital of Ningxia Medical University, Yinchuan, Ningxia, China; 2 State Key Laboratory of Pathogenesis, Prevention and Treatment of High Incidence Diseases in Central Asia, Xinjiang Medical University, Ürümqi, China; 3 Ningxia Key Laboratory of Clinical and Pathogenic Microbiology, General Hospital of Ningxia Medical University, Yinchuan, Ningxia, China; 4 School of Clinical Medicine, Ningxia Medical University, Yinchuan, Ningxia, China

**Keywords:** Diquat (1,1′-ethylene-2,2′-bipyridyl, DQ), mitochondrial dysfunction, reactive oxygen species (ROS), regulated cell death, targeted therapy

## Abstract

Diquat (1,1′-ethylene-2,2′-bipyridyl, DQ) is a herbicide widely used for weed control in both agricultural and non-cultivated areas. Although its acute toxicity is lower than that of paraquat, its high-water solubility and stability in acidic and neutral environments contribute to its prolonged environmental persistence. As DQ gradually replaces paraquat in agricultural practice, the incidence of DQ poisoning has increased significantly. DQ poisoning typically results from accidental ingestion, suicidal intake, or improper agricultural handling. To date, no specific antidote is available, and the high mortality associated with DQ poisoning presents a critical challenge for clinical management. Accumulating evidence indicates that the toxicity of DQ is primarily attributed to its capacity to generate reactive oxygen species (ROS), leading to oxidative stress and subsequent oxidative damage to lipids, proteins, and DNA, ultimately resulting in multi-organ dysfunction, with the kidneys and intestines being the primary target organs. The pathogenesis of DQ poisoning involves multiple factors, including oxidative stress imbalance, regulated cell death, mitochondrial dysfunction, and disturbances in energy metabolism. This review systematically examines the physicochemical properties, metabolic characteristics, biodistribution, and target organ toxicity of DQ, with a particular focus on the interplay between excessive ROS production and mitochondrial dysfunction in the context of oxidative stress. Furthermore, we provide an in-depth discussion on the roles of regulated cell death—including pyroptosis, ferroptosis, and mitophagy—and metabolic dysregulation in DQ-induced toxicity. In addition, this review summarizes the classical signaling pathways involved in organ dysfunction, current therapeutic strategies, and potential intervention targets, thereby offering a theoretical framework and future research directions for the management of DQ poisoning.

## 1 Introduction

Diquat (1,1′-Ethylene-2,2′-bipyridinium, DQ) is a widely utilized bipyridinium herbicide in both agricultural and non-cultivated land for effective weed management, owing to its rapid onset of action and broad-spectrum herbicidal activity (Ren et al., 2024). Although Diquat degrades into simple organic compounds and inorganic salts under alkaline conditions, its high water solubility and stability in neutral and acidic environments result in a prolonged environmental persistence (Guo et al., 2023). Since the initial report of Diquat poisoning by Oreoopoulos et al., in 1968, the incidence of poisoning cases has progressively increased globally. This trend has been particularly notable with the gradual replacement of Paraquat (PQ) by Diquat in agricultural practices, which has led to a significant rise in Diquat-related toxicological incidents (Yin et al., 2015). Despite its relatively lower acute toxicity in comparison to Paraquat, Diquat presents considerable ecological risks due to its bioaccumulation and persistence in soil and aquatic ecosystems (Akhavein and Linscott, 1968). Diquat poisoning is primarily associated with accidental ingestion or suicidal ingestion, although unintentional exposure due to improper agricultural handling also contributes to cases of poisoning. At present, there is no established antidote for Diquat toxicity, and the high mortality rate associated with Diquat poisoning continues to pose a substantial clinical challenge. To improve the clinical prognosis of patients suffering from Diquat poisoning, it is imperative to elucidate the underlying pathogenic mechanisms and develop targeted therapeutic interventions.The toxicity of Diquat is primarily attributed to its redox cycling capabilities, which facilitate the generation of reactive oxygen species (ROS) such as superoxide anions (O_2_•^−^) and hydroxyl radicals (OH·) (Ren et al., 2024), (Wen et al., 2020).These ROS instigate oxidative damage to lipids, proteins, and DNA, thereby triggering multi-organ dysfunction. The kidneys are recognized as the principal target organs in Diquat toxicity, with the intestines being among the most susceptible organs. Acute tubular necrosis (ATN) is a hallmark feature of Diquat-induced renal damage, and common clinical manifestations include elevated blood urea nitrogen (BUN) levels, increased creatinine, reduced glomerular filtration rate (GFR), and decreased urine output. However, the precise pathophysiological mechanisms underlying Diquat-induced acute renal injury, intestinal damage, and other target organ dysfunctions remain incompletely characterized. Available research indicates that mechanisms such as oxidative stress imbalance, regulatory cell death, mitochondrial dysfunction, and disruptions in energy and metabolic homeostasis are likely involved in the pathogenesis of Diquat toxicity. Nevertheless, these studies remain fragmented and necessitate further integration and comprehensive evaluation. This review seeks to comprehensively examine the physicochemical properties, metabolic characteristics, pharmacokinetics, and target organ damage associated with Diquat. It will further explore the shared pathogenic mechanisms of organ dysfunction resulting from Diquat exposure, with a particular focus on the interplay between oxidative stress-mediated ROS overproduction and mitochondrial dysfunction in the context of regulatory cell death and metabolic disturbances. Moreover, this review will synthesize current research on well-established signaling pathways involved in organ dysfunction and discuss prevailing therapeutic approaches and potential novel therapeutic targets for Diquat poisoning.

## 2 Physicochemical properties of DQ

DQ is a bipyridinium herbicide that is typically formulated and used as its dibromide salt (C_12_H_12_Br_2_N_2_, molecular weight 344.1 g/mol), which predominantly exists as a monohydrate in the pure state (Kim et al., 2025; Magalhães et al., 2018). The pure dibromide salt of DQ is a yellow crystalline compound that is devoid of odor, while commercial formulations are typically presented as deep brown, transparent liquids, often accompanied by an earthy odor (Magalhães et al., 2018). As a permanently charged quaternary ammonium cation (DQ^2+^), DQ is fully ionized in aqueous solution and behaves as a strong electrolyte (Kim et al., 2025; Magalhães et al., 2018). The compound displays significant water solubility (700 g/L at 20 °C) and is stable in acidic and neutral pH environments; however, it undergoes rapid hydrolysis under alkaline conditions (He et al., 2025; Wang P. et al., 2024; Simsiman et al., 1976). In terms of its physical properties, DQ has an extremely low vapor pressure (less than 0.00001 mmHg at 20 °C), indicating negligible volatility under environmental conditions (Kim et al., 2025; Magalhães et al., 2018). The density of the dibromide salt ranges from 1.22 to 1.27 g/cm^3^ (20 °C) (Kim et al., 2025; Magalhães et al., 2018). The environmental behavior of DQ is strongly influenced by its high-water solubility and ionic nature. The octanol–water partition coefficient (log Kow) has been estimated to be approximately 2.36 (for the neutral form) by computational models; however, it is important to note that DQ is a dication under physiological and environmental conditions (log Kow ≈ −4.6 for the ionized form), which profoundly reduces its lipophilicity and limits passive diffusion across biological membranes (Kim et al., 2025; Magalhães et al., 2018). Consequently, DQ is effectively retained in the aqueous phase and does not readily partition into organic matrices.

The double positive charge on the diquat cation fundamentally drives its unique environmental fate and toxicokinetics. In terrestrial ecosystems, DQ exhibits an exceptionally strong affinity for negatively charged soil components, particularly clay minerals and organic matter, through electrostatic interactions, resulting in extensive adsorption and profoundly limiting its mobility, vertical leaching, and bioavailability (Kim et al., 2025; Magalhães et al., 2018; He et al., 2025). Consequently, DQ remains primarily confined to the upper layers of the soil and is unlikely to contaminate groundwater. The adsorbed DQ is largely biologically and chemically inactive, yet residues have been shown to persist in soil for extended periods (Magalhães et al., 2018; He et al., 2025; Wang P. et al., 2024; Simsiman et al., 1976). In aquatic environments, DQ undergoes photodegradation and microbial breakdown, with a half-life ranging from 2 to 10 days, and is also removed through adsorption to suspended particles and sediments (He et al., 2025; Wang P. et al., 2024; Simsiman et al., 1976). These inherent physicochemical properties, specifically the high water solubility, extremely low volatility, strong soil adsorption due to the dicationic structure, and marked stability under neutral and acidic conditions, render DQ both environmentally persistent in soil and potentially ecotoxic, with significant implications for its long-term ecological footprint.

DQ predominantly exists in the form of its dibromide salt, with commercial formulations typically containing a 20% (w/v) concentration (Expert Consensus Group on the Diagnosis and Treatment of Acute Diquat Poisoning, 2020). The pure dibromide salt of DQ is a yellow crystalline compound that is devoid of odor and exhibits high solubility in water under standard conditions (Magalhães et al., 2018). In contrast, commercial formulations of DQ are typically presented as deep brown, transparent liquids, often accompanied by an earthy odor (Magalhães et al., 2018). As a bipyridinium herbicide, DQ in its pure form is a yellow crystalline solid, odorless, while commercial variants are deep green to reddish-brown. DQ has a water solubility of 712 g/L at pH 5.2 and is stable in acidic and neutral environments but hydrolyzes under alkaline conditions (Wang P. et al., 2024; Simsiman et al., 1976). It exhibits considerable environmental persistence, particularly in aquatic systems (half-life 2–10 days), where it degrades into simple organic compounds and inorganic salts. These properties contribute to its environmental residue and potential ecotoxicity, with implications for long-term ecological impact.

## 3 Environmental forms and metabolism of DQ

The environmental behavior and transformation of DQ are predominantly governed by the medium in which it is situated. In terrestrial ecosystems, DQ exhibits a strong affinity for adsorption to soil particles, which substantially limits its mobility and leads to a prolonged degradation process (Kim et al., 2025). In soils, DQ degradation is primarily facilitated through microbial and chemical pathways, with microbial metabolism playing a critical role in its breakdown (Kim et al., 2025). In aquatic environments, DQ is removed *via* photodegradation and microbial activity, particularly after it binds to suspended particulate matter (Lopes and Manso, 1989). While the degradation of DQ in water is relatively rapid, the resultant degradation products may still pose a risk of secondary toxicity to aquatic organisms, highlighting the potential for cumulative environmental impacts. Moreover, due to its bioaccumulative nature, DQ has the potential to persist within the ecosystem, representing a long-term ecological risk to aquatic biota and broader environmental health. These attributes underscore the necessity for comprehensive assessments of the compound’s environmental persistence and the potential hazards associated with its breakdown products (Lopes and Manso, 1989).

## 4 Distribution, absorption, and primary target organ damage of DQ in the body

DQ is primarily absorbed *via* the gastrointestinal tract, with its bioavailability being relatively low (<10%) upon oral administration (Duan et al., 2024). Following absorption, DQ is actively transported into various tissues through diffusion or ion pump-mediated processes, resulting in rapid systemic distribution (Wu J. et al., 2024; McCarthy and Speth, 1983). The gastrointestinal tract serves as the primary entry point, although its absorption can be influenced by the presence of certain foods and gut microbiota, which can partially degrade the compound and mitigate its absorption. While DQ exhibits minimal percutaneous absorption and is poorly absorbed *via* the respiratory tract, the skin acts as a significant barrier. However, this barrier function is compromised when the skin is damaged, leading to increased absorption under such conditions (Magalhães et al., 2018; Expert Consensus Group on the Diagnosis and Treatment of Acute Diquat Poisoning, 2020). Upon systemic distribution, DQ rapidly accumulates in multiple organs, with peak concentrations observed in the liver, kidneys, gastrointestinal tract, and lungs within 2 hours of administration (Magalhães et al., 2018). Subsequently, the concentrations decline rapidly over time. Notably, DQ induces mild and reversible damage to type I pulmonary cells, contrasting with PQ, which exerts more severe damage to both type I and type II alveolar cells (Wu et al., 2022). This indicates that, while the lungs are affected, they are not the primary target organ of DQ toxicity (Wu et al., 2022).

Regarding its metabolic processing, DQ is relatively stable in the body, undergoing limited biotransformation primarily through cytochrome P450 enzymes in the liver (Yin et al., 2015; Chen J. et al., 2021). This biotransformation leads to the production of less toxic metabolites, such as monopyridone and dipyridone derivatives (Yin et al., 2015; Chen J. et al., 2021). The majority of DQ (approximately 94%) is excreted unchanged in the feces within 24 h post-ingestion, reflecting its limited gastrointestinal absorption (He et al., 2025). Approximately 45% of the absorbed DQ is eliminated *via* the urine within 48 h, with trace amounts excreted in the bile (He et al., 2025).

**FIGURE 1:**
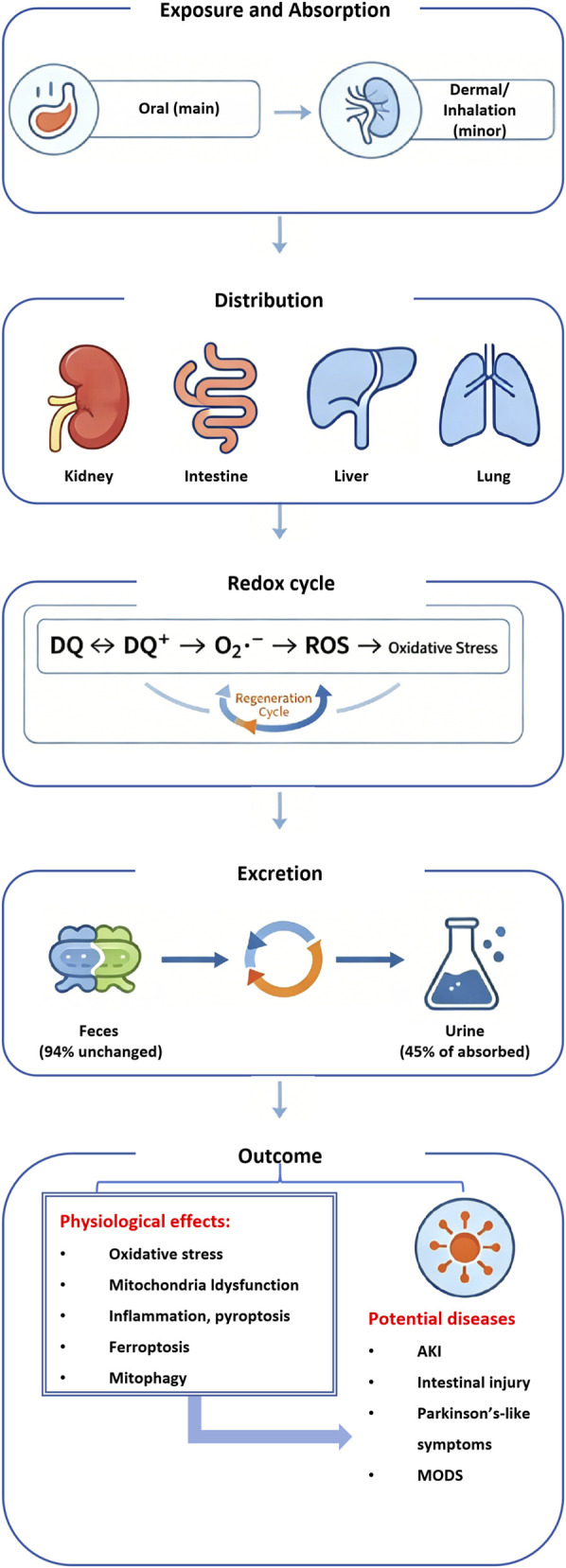
Schematic overview of the absorption, distribution, redox cycling, excretion, and downstream physiological effects and potential diseases induced by Diquat (DQ) toxicity. DQ is primarily absorbed *via* the oral route (with minor dermal/inhalation routes) and distributes mainly to the kidneys, intestines, liver, and lungs. Inside cells, DQ undergoes a redox cycle (DQ ⇄ DQ^+^ → O_2_^•-^ → ROS → oxidative stress), with a regeneration cycle that sustains ROS production. DQ is excreted largely unchanged in feces (94%) and, of the absorbed fraction, 45% in urine. The sustained oxidative stress leads to multiple physiological effects, including mitochondrial dysfunction, inflammation, pyroptosis, ferroptosis, and mitophagy, ultimately contributing to potential diseases such as acute kidney injury (AKI), intestinal injury, Parkinson’s disease-like non-motor symptoms, and multiple organ dysfunction syndrome (MODS).

Post-mortem analyses of patients who died from acute DQ poisoning reveal varying concentrations of DQ in different tissues and bodily fluids, depending on the elapsed time since exposure. In one fatal case of DQ poisoning, the highest concentration of DQ was detected in the urine 14 h after death, followed by vitreous humor, lungs, liver, brain, and kidneys (McCarthy and Speth, 1983). During metabolism, DQ undergoes a redox cycling process, generating highly reactive DQ cations (DQ^+^), which subsequently interact with molecular oxygen to form superoxide anions (O_2_•^-^) and other reactive oxygen species (ROS). These ROS are central to the induction of cellular damage, contributing to lipid peroxidation, protein oxidation, and DNA damage (Gou et al., 2024; Yang et al., 2023; Jiang et al., 2025). Ultimately, this leads to oxidative stress, disruption of cellular membranes, mitochondrial dysfunction, and the initiation of apoptotic pathways (Gou et al., 2024; Yang et al., 2023; Jiang et al., 2025) (Figure 1). Despite its relatively inefficient excretion, DQ persists in the body, maintaining its toxic effects primarily through urinary and fecal elimination, with most of the compound excreted in its parent form (Magalhães et al., 2018; Basilicata et al., 2022; Jones and Vale, 2000). DQ has been implicated in the induction of systemic oxidative stress and inflammatory responses, which contribute to the pathogenesis of multiple organ dysfunction syndrome (MODS) (Yoshioka et al., 1992; Huang et al., 2021). The specific mechanisms of DQ toxicity in target organs, including the renal system, involve oxidative stress, aberrant activation of inflammatory signaling pathways, dysregulation of autophagy, and metabolic disturbances (Wu et al., 2022; Xia et al., 2023; Rogers et al., 2006). While significant advances have been made in understanding the toxicity of DQ, existing research remains fragmented, necessitating further integration and clarification of these complex mechanisms. This review aims to provide an in-depth exploration of DQ’s direct nephrotoxic effects and its broader impact on systemic metabolic processes, with an emphasis on elucidating underlying toxicological pathways and identifying potential therapeutic strategies for mitigating toxicity.

## 5 Mechanisms of DQ in target organ dysfunction

DQ predominantly traverses cellular membranes *via* passive diffusion, with a lesser degree of active transport mediated by cation pumps (Magalhães et al., 2018). Upon exposure to renal cells, DQ exerts direct cytotoxic effects, initially interacting with the renal mucosa and resulting in widespread tissue damage. Simultaneously, a significant release of pro-inflammatory mediators occurs, which can instigate both direct and indirect injury to renal tissue, thereby facilitating further absorption of DQ (Huang et al., 2023). Recent single-cell RNA sequencing analyses have identified that DQ induces an oxidative stress-driven microenvironment within endothelial and renal parenchymal cells (Chen et al., 2025). This microenvironment is typified by the activation of oxidative phosphorylation pathways and an amplified inflammatory response (Chen et al., 2025). The resulting oxidative stress leads to the modulation of cell death mechanisms in both parenchymal and immune cells, stimulating the release of pro-inflammatory cytokines and exacerbating the existing oxidative milieu. This metabolic shift impairs hepatic detoxification functions and leads to a reprogramming of immune cell activation, further contributing to the exacerbation of tissue damage.

### 5.1 Mitochondrial dysfunction and oxidative stress in the pathogenesis of DQ toxicity

**FIGURE 2:**
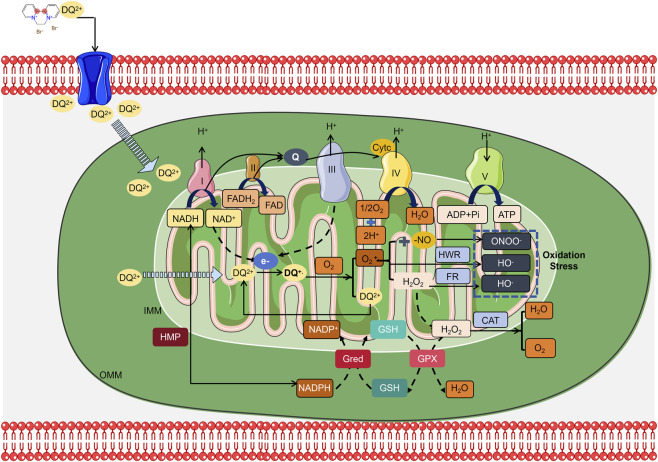
The process of intracellular redox cycling and reactive species production induced by DQ. Upon entering the cell, DQ undergoes a redox cycle, generating highly reactive DQ cations (DQ^+^), which further combine with molecular oxygen to form superoxide anions (O_2_^•-^) and other ROS. SOD: Superoxide Dismutase; NADPH: Nicotinamide Adenine Dinucleotide Phosphate (reduced); HMP: Hexose Monophosphate Pathway; CAT: Catalase; GPX: Glutathione Peroxidase; Gred (or GR): Glutathione Reductase; CYP450: Cytochrome P450; FR: Fenton Reaction; HWR: Haber-Weiss Reaction.

Extensive research on pesticide toxicity has demonstrated that nearly all pesticides, including DQ, can induce oxidative stress, which is characterized by an imbalance between the generation of ROS and the capacity of cellular defense mechanisms to neutralize excess ROS (Bhattacharyya et al., 2014; Li W. et al., 2022; Hajhashemi et al., 2010). Under physiological conditions, a delicate balance is maintained between oxidants and antioxidants within biological systems (Lightfoot et al., 2006). However, when ROS are produced in excess, they can induce cellular damage by targeting proteins, lipids, and DNA, leading to irreversible cellular injury and death (Komatsu et al., 2020). While DQ does not directly form covalent bonds with macromolecules such as lipids, proteins, or nucleic acids, it triggers oxidative stress through redox cycling mechanisms, resulting in the generation of reactive oxygen and nitrogen species. Notably, DQ possesses a high redox potential, which confers its strong ability to generate ROS. Upon entering the organism, DQ accepts an electron from NADPH *via* the action of NADPH II and cytochrome P450 (CYP450) reductases, leading to the reduction of DQ and the formation of NADP^+^ along with an unstable DQ radical (DQ^+^). The DQ^+^ radical subsequently transfers an electron to molecular oxygen, thereby generating superoxide anion radicals (O_2_•^-^), while DQ returns to its original form. This cycle repeats, resulting in the continuous generation of oxygen-derived ions, which, either spontaneously or catalytically *via* superoxide dismutase (SOD), lead to the formation of hydrogen peroxide (H_2_O_2_) and other ROS. Under normal circumstances, ROS are neutralized and converted to water by enzymes such as catalase and glutathione peroxidase. However, when ROS production exceeds the body’s antioxidant and compensatory capacity, oxidative stress ensues (Figure 2). This process predominantly occurs within the mitochondria, rendering mitochondrial dysfunction and oxidative stress imbalance as central mechanisms in DQ-induced toxicity.

Several studies have directly demonstrated that DQ exposure induces mitochondrial dysfunction in various experimental models. For instance, in weaned piglets challenged with DQ, Cao et al. reported that DQ significantly reduced the activities of mitochondrial complexes I-IV in the jejunum, accompanied by decreased ATP levels and increased mitochondrial ROS production (Cao et al., 2018). Similarly, in a rat model of acute DQ poisoning, Chen et al. observed that DQ triggered mitochondrial swelling, loss of mitochondrial membrane potential (ΔΨm), and activation of the mitochondrial apoptotic pathway in the liver (Chen J. et al., 2021). In renal proximal tubular cells, Wu et al. showed that DQ caused mitochondrial fragmentation, decreased mitochondrial membrane potential, and impaired ATP synthesis, all of which were ameliorated by CoQ10 supplementation (Wu J. et al., 2024). Moreover, in *Caenorhabditis elegans*, Wang et al. found that DQ induced a marked downregulation of mitochondrial complex II and upregulation of complex III, leading to excessive mitochondrial ROS generation (Wang B. et al., 2025). These findings collectively confirm that mitochondrial dysfunction is a direct and early event in DQ toxicity, not merely a consequence of oxidative stress. Therefore, preserving mitochondrial integrity and function represents a promising therapeutic strategy against DQ-induced organ injury.

#### 5.1.1 DQ-induced pyroptosis in organ dysfunction

Pyroptosis is a form of programmed cell death mediated by gasdermin proteins, characterized by the formation of membrane pores, release of cellular contents, and activation of inflammatory responses (Su et al., 2023). This process plays a crucial role in inflammation. The morphological hallmarks of pyroptosis include cell swelling, the formation of bubble-like protrusions, and the development of pyroptotic bodies, distinguishing it from apoptosis and necrosis. Pyroptosis is triggered by the activation of inflammasomes, which in turn activate inflammatory caspases such as caspase-1, caspase-4, caspase-5, and caspase-11. These caspases cleave gasdermin proteins, leading to the formation of membrane pores and ultimately resulting in cell lysis.

ROS directly impair the integrity of the cell membrane, increasing membrane permeability and facilitating the formation of membrane pores mediated by gasdermins, thus accelerating the pyroptotic process. The administration of DQ induces an excessive production of ROS within the body. ROS directly impair the integrity of the cell membrane, increasing membrane permeability and facilitating the formation of membrane pores mediated by gasdermins, thus accelerating the pyroptotic process. Furthermore, ROS can activate the non-canonical pyroptotic pathway, which involves caspases-4/5/11, further enhancing pyroptosis. In addition, excessive ROS can trigger the activation of the NLRP3 inflammasome in DQ-exposed models. However, the precise molecular mechanism by which DQ-induced ROS activates NLRP3, such as the dissociation of thioredoxin (TRX) from thioredoxin-interacting protein (TXNIP) reported in other disease contexts (e.g., diabetic nephropathy), has not yet been directly demonstrated in DQ toxicity and warrants further investigation. Once activated, the NLRP3 inflammasome recruits caspase-1, which cleaves gasdermin D (GSDMD), leading to the formation of membrane pores, the release of cellular contents, and the secretion of inflammatory cytokines, thereby initiating pyroptosis. Direct evidence supporting DQ-induced pyroptosis has been reported in several recent studies (Chen et al., 2024; Qiao et al., 2022; Wang K. et al., 2025).

**FIGURE 3:**
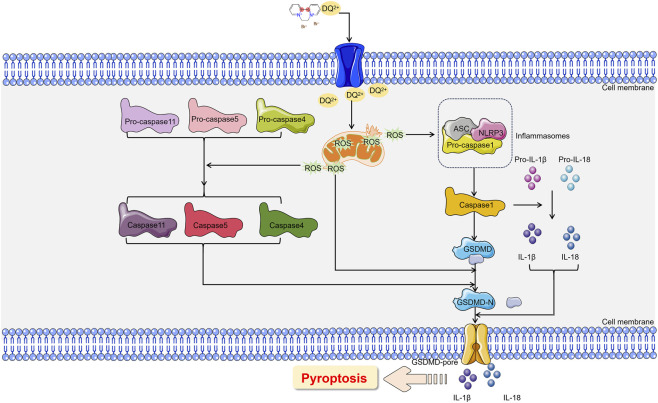
Schematic representation of the pyroptosis pathway induced by DQ poisoning. During DQ exposure, excessive ROS production triggers mitochondrial dysfunction and inflammasome activation. Pyroptosis proceeds *via* caspase-1-dependent (NLRP3 inflammasome→caspase-1→GSDMD cleavage) and caspase-1-independent (caspase-4/5/11 → GSDMD cleavage) pathways, leading to pore formation, release of IL-1β/IL-18, and inflammatory cell death. ASC: Apoptosis-associated speck-like protein containing a CARD; NLRP3: Nucleotide-binding oligomerization domain, leucine-rich repeat, and pyrin domain-containing 3; GSDMD: Gasdermin D.

The pyroptotic pathway induced by DQ toxicity can be classified into two distinct routes based on caspase dependency: the caspase-1-dependent and caspase-1-independent pyroptosis pathways (Zhang et al., 2024). In the caspase-1-dependent pyroptosis pathway, the invasion of cells by various pathogens triggers the activation of inflammasomes, including NLRP3, NLRC4, AIM2, and Pyrin, which detect these signals. Upon activation, these inflammasomes recruit ASC, which in turn associates with pro-caspase-1, leading to the activation of caspase-1 (Rao et al., 2022). Activated caspase-1 cleaves gasdermin D (GSDMD), exposing its N-terminal domain, which then interacts with phospholipids on the cell membrane, forming pores and facilitating the release of cellular contents, thus initiating pyroptosis. In addition, caspase-1 cleaves the precursors of interleukins IL-1β and IL-18, converting them into their active forms. These mature cytokines are subsequently released into the extracellular space, further amplifying the inflammatory response (Yu et al., 2021). The caspase-1-independent pyroptosis pathway operates through an alternative mechanism: Upon exposure to lipopolysaccharide (LPS), human caspases-4/5 and murine caspase-11 directly bind to LPS, becoming activated and cleaving GSDMD. This cleavage exposes the N-terminal of GSDMD, thereby initiating the pyroptotic process. Additionally, activated caspase-4/5/11 can induce the activation of the Pannexin-1 channel, which results in the efflux of potassium ions (K^+^) into the extracellular environment. This potassium release activates the NLRP3 inflammasome, leading to the activation of caspase-1, thereby triggering the caspase-1-dependent pyroptosis pathway and amplifying the overall pyroptotic response (Liu et al., 2016) (Figure 3).

Chen et al. demonstrated that exposure to DQ leads to the accumulation of mitochondrial ROS in an HK2 cell model, which subsequently triggers the cleavage of gasdermin E (GSDME) *via* the mitochondrial intrinsic apoptotic pathway (Chen et al., 2024). Notably, the knockout of GSDME mitigated the pyroptotic cell death induced by DQ. Previous studies have indicated that Nrf2 plays a protective role by suppressing the activation of the NLRP3 inflammasome, thereby reducing the release of key pro-inflammatory cytokines, including IL-6, IL-18, IL-1β, and TNF-α (Liu et al., 2018). In line with these findings, Qiao et al. confirmed that DQ exposure significantly elevated the levels of the NLRP3 inflammasome in mice, which, in turn, facilitated the increased production of IL-18 and IL-1β (Qiao et al., 2022). Moreover, in an oxidative stress model induced by DQ, a marked increase in the concentrations of IL-6 and TNF-α was observed in piglets, underscoring the pivotal role of TNF-α as a pro-inflammatory mediator in systemic inflammation (Yuan et al., 2017). KEGG pathway analysis of differentially expressed genes (DEGs) in mice exposed to DQ highlighted significant enrichment in the NF-κB signaling pathway (Yuan et al., 2024). This observation was further substantiated by additional research. IL-17 receptors, predominantly expressed in the spleen and kidneys, are implicated in triggering inflammatory responses upon ligand binding. DQ-induced oxidative stress elevates IL-17 levels, which activates the NF-κB signaling cascade, leading to the upregulation of downstream inflammatory mediators such as IL-6 and TNF-α, thereby amplifying the inflammatory response (Chen et al., 2019). Furthermore, IL-17 significantly upregulates TLR3 expression in renal tissues, and the activation of NF-κB by TLR3 exacerbates renal inflammation and cell death, contributing to DQ-induced acute kidney injury (Fussell et al., 2011). Beyond renal toxicity, recent studies have revealed that DQ exposure can activate the TLR4/NF-κB/NLRP3 signaling axis, resulting in neurodegenerative processes in dopaminergic neurons and the development of Parkinson’s disease-like non-motor symptoms in mice (Wang K. et al., 2025).

In conclusion, DQ triggers excessive ROS production, which compromises the integrity of kidney cell membranes, leading to pore formation mediated by GSDMD and thereby accelerating pyroptosis. Additionally, DQ induces mitochondrial dysfunction and activates the NLRP3 inflammasome, culminating in pyroptotic cell death and subsequent renal damage. Thus, targeting oxidative stress-induced pyroptosis could emerge as a potential therapeutic approach to mitigate renal injury associated with DQ exposure.

#### 5.1.2 DQ-induced autophagy in organ dysfunction

**FIGURE 4:**
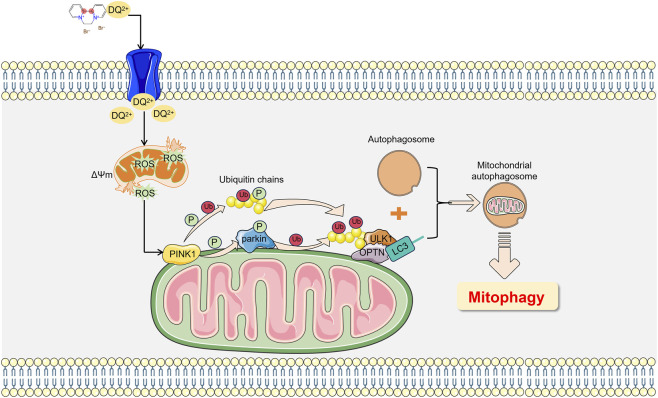
Molecular mechanism of mitophagy activation following DQ exposure. DQ-induced oxidative stress causes mitochondrial membrane potential depolarization, leading to PINK1 accumulation on the OMM. PINK1 recruits and activates Parkin, which ubiquitinates OMM proteins. The ubiquitin chains (represented by solid yellow spheres) recruit autophagy receptors (e.g., OPTN) and LC3, resulting in autophagosome formation and mitophagy. OMM,outer mitochondrial membrane; OPTN, Optineurin; ULK1, UNC-51-like kinase 1; LC3, light chain 3.

As discussed above, DQ-induced oxidative stress causes mitochondrial damage (Wang B. et al., 2025; Chen et al., 2024; Li et al., 2022a). In response, cells activate mitophagy, a selective autophagic process that removes dysfunctional mitochondria to maintain homeostasis (Wu J. et al., 2024; Remex et al., 2024). Upon exposure to harmful agents, compromised mitochondria release excessive ROS, thereby exacerbating oxidative injury (De Gaetano et al., 2021; Sweetman et al., 2020; Li et al., 2022b). Autophagy, a cellular process in which damaged proteins and organelles are sequestered within double-membrane vesicles (autophagosomes) and degraded in the lysosome, is an essential mechanism for maintaining cellular homeostasis and regulating cell death (Yamamoto and Matsui, 2024). Mitochondrial autophagy, or mitophagy, is activated under conditions of severe stress, elevated ROS levels, and the accumulation of dysfunctional mitochondria, with key regulatory factors including BCL2 family proteins, Parkin, and BNIP3 (Wang and Zhou, 2020). The principal mechanism underlying mitophagy involves the activation of the PINK1/Parkin pathway, where PINK1 enhances the process by modulating the levels of adenosine triphosphate (ATP) dynamics (Hertz et al., 2013) (Figure 4).

Research on DQ-induced mitochondrial autophagy has predominantly focused on intestinal injury. It has been shown that exposure to DQ leads to the accumulation of ROS, which subsequently disrupts the structural integrity and functional capacity of mitochondrial DNA (mtDNA) and mitochondrial enzymes, resulting in mitochondrial dysfunction in the jejunal tissue of weaned piglets (Ballard and Towarnicki, 2020; Chen et al., 2021). The dysfunction of damaged mitochondria further amplifies the production of mitochondrial ROS (mtROS), establishing a detrimental feedback loop. Furthermore, the accumulation of mtROS has been demonstrated to reduce the diversity of gut microbiota and impair the host’s antimicrobial defense mechanisms (Ballard and Towarnicki, 2020; Yardeni et al., 2019).

Oxidative stress is a well-established trigger of mitochondrial autophagy, and DQ-induced oxidative stress has been shown to impair intestinal epithelial function, accompanied by mitochondrial dysfunction in the jejunal mucosa and the activation of mitochondrial autophagy (Yin et al., 2015; Xu et al., 2022). Mitochondrial autophagy, by engulfing and degrading damaged mitochondria, aids in the clearance of excess ROS, thus serving a protective role in cellular homeostasis (Saita et al., 2013; Prashar et al., 2024; Alan et al., 2022; Kim and Lemasters, 2011). The PINK1/Parkin signaling pathway is a canonical mechanism of mitochondrial autophagy, and oxidative stress has been shown to activate this pathway, thereby inducing mitochondrial autophagy (Zhang et al., 2021). In addition, research indicates that DQ exposure induces the formation of autophagic vesicles and upregulates the expression of proteins involved in mitochondrial autophagy, further suggesting the activation of this pathway (Wen et al., 2024). Recent studies emphasize the pivotal protective role of mitochondrial autophagy in mitigating cell death caused by DQ-induced oxidative stress (Cao et al., 2018). This process involves the selective sequestration of damaged mitochondria by autophagosomes, which are subsequently degraded in the lysosome, thereby preserving the normal function of intestinal epithelial cells and reducing further oxidative damage (Cao et al., 2018).

As previously noted, Nrf2 attenuates oxidative stress by regulating the transcription of antioxidant genes, while mitochondrial autophagy mitigates damage by selectively eliminating dysfunctional mitochondria. The cooperative interplay of these two mechanisms contributes to cellular homeostasis and facilitates the repair of oxidative damage. Consequently, mitochondrial autophagy may offer an effective approach to ameliorate organ damage, particularly intestinal injury, induced by DQ exposure. Wang et al. established a diquat-challenged pig model, demonstrating that DQ injection induced severe intestinal oxidative stress (Wang C. et al., 2019) Treatment with butyrate, through selective activation of mitochondrial autophagy, markedly enhanced the expression of PINK1 and Parkin proteins in mitochondria, leading to a reduction in oxidative stress and inflammation within the intestines, as well as an improvement in mitochondrial functio (Wang C. et al., 2019). In a separate study, Lee et al. observed that NaBu treatment resulted in a progressive decline in mitochondrial membrane potential and quality, accompanied by the accumulation of the mitochondrial autophagy protein Parkin on the mitochondria. These findings suggest that the protective role of autophagy may be mediated by the removal of damaged mitochondria, which ultimately contributed to increased survival rates in hamster ovarian cells (Lee and Lee, 2012). Further investigations have demonstrated that butyrate can enhance mitochondrial function under physiological stress and/or mitochondrial dysfunction, positioning it as a potentially critical metabolite that aids in the restoration of energy metabolism in disease contexts (Rose et al., 2018).

#### 5.1.3 DQ-induced ferroptosis in organ dysfunction

In addition to pyroptosis and mitophagy, emerging evidence indicates that ferroptosis, an iron-dependent form of regulated cell death, plays a critical role in DQ-induced organ damage, particularly in the kidneys and intestines (Prasad et al., 1987). This phenomenon is closely linked to the compound’s potent redox activity and cytotoxicity (Prasad et al., 1987). Diquat promotes excessive ROS production, leading to mitochondrial dysfunction and cellular damage (Drechsel and Patel, 2009). In a diquat-induced zebrafish poisoning model, an inflammatory response characterized primarily by neutrophil infiltration was observed (Huang et al., 2023; Qu et al., 2024). The interplay of oxidative stress and inflammation in acute kidney injury is complex; however, accumulating evidence suggests that ferroptosis plays a pivotal role in this process. Specifically, diquat exposure has been shown to cause jejunal tissue damage and impair barrier function in piglets, with underlying mechanisms involving oxidative stress-induced apoptosis and ferroptosis (Liu et al., 2024). *In vitro* studies further support that diquat disrupts mitochondrial genomic stability in endothelial cells and mediates cellular injury *via* ZBP1/RIPK3-dependent necroptosis and ferroptosis pathways, highlighting potential therapeutic targets (Chen et al., 2024). Moreover, DQ exposure induces excessive accumulation of mitochondrial ROS, downregulates ferritin heavy chain 1 (FTH1), and causes Fe^2+^ overload, all of which contribute to ferroptosis-related damage in renal tissue (Dolma et al., 2003). Ferroptosis is an iron-dependent form of regulated cell death, typically occurring in environments characterized by metabolic disturbances and oxidative stress (Tang et al., 2019; Dixon et al., 2012). Under normal conditions, the oxidative system composed of iron ions, the Fenton reaction, and ROS is counterbalanced by an antioxidant defense system involving the cystine/glutamate antiporter (system Xc^−^), GPX4, and glutathione (GSH) (system Xc^−^/GSH/GPX4 axis), along with other recently identified pathways that maintain cellular and systemic homeostasis. During DQ-induced toxicity, renal tubular epithelial cells are particularly vulnerable to oxidative stress, making them prone to ferroptosis, which ultimately leads to structural damage of the renal tubules, reduced glomerular filtration rate, and the onset of acute kidney injury (Zhu et al., 2019). In summary, ferroptosis is a central mechanism underlying DQ-induced renal injury and other targeted organ dysfunction. Elucidating this mechanism is crucial for the development of targeted intervention strategies.

**FIGURE 5:**
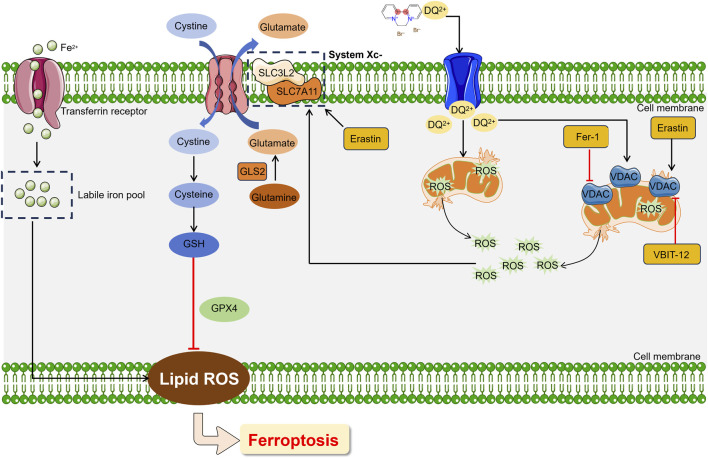
Key molecular pathways of ferroptosis in DQ-induced renal and organ injury. Exogenous, endogenous, and VDAC-mediated ferroptosis pathways triggered by DQ toxicity. The exogenous pathway involves inhibition of System Xc^-^, reducing cystine uptake and GSH synthesis, thereby inactivating GPX4. The endogenous pathway is driven by GPX4 inhibition or loss. The VDAC pathway involves DQ-induced upregulation of VDAC, leading to mitochondrial ROS leakage and iron overload. Inhibitors such as Fer-1 and VBIT-12 block these pathways. GSH, Glutathione; GPX4, Glutathione peroxidase 4; GLS2, Glutaminase2; VDAC, Voltage-dependent anion channel.

Ferroptosis is an iron-dependent form of regulated cell death characterized by lipid peroxidation and intracellular iron overload (Zhu et al., 2019; Wang Y. et al., 2024). In DQ toxicity, ferroptosis has been linked to three primary pathways: the exogenous (transport protein-dependent) pathway involving system Xc-, the endogenous (enzyme-regulated) pathway involving GPX4, and the voltage-dependent anion channel (VDAC) pathway (Bergmann et al., 2009; Jing et al., 2025; Chen X. et al., 2021). Although the detailed molecular mechanisms of these pathways have been extensively characterized in other disease models, direct evidence in DQ-exposed systems is still emerging. Specifically, in DQ-treated piglets and cell models, DQ exposure has been shown to cause excessive mitochondrial ROS accumulation, downregulate ferritin heavy chain 1 (FTH1), and induce Fe^2+^ overload, all of which contribute to ferroptosis-related damage (Dolma et al., 2003). In a zebrafish model, DQ significantly upregulated VDAC1 and mitochondrial ferritin (FTMT), and the ferroptosis inhibitor Fer-1 reversed these changes (Ou et al., 2025). Furthermore, *in vitro* studies demonstrated that DQ disrupts mitochondrial genomic stability and mediates cell injury *via* ZBP1/RIPK3-dependent necroptosis and ferroptosis pathways (Chen et al., 2024). These findings collectively indicate that ferroptosis is a central mechanism in DQ-induced renal and other organ damage. A schematic representation of the proposed ferroptosis pathways in DQ toxicity is provided in Figure 5.

VDAC (voltage-dependent anion channel) is a mitochondrial outer membrane pore protein that regulates the transport of ions and metabolites. In the context of DQ toxicity, VDAC1 expression is significantly upregulated, as demonstrated in a zebrafish model (Ou et al., 2025). Inhibition of ferroptosis with Fer-1 or the VDAC1 inhibitor VBIT-12 reduces VDAC1 oligomerization, restores GPX4 levels, and alleviates mitochondrial damage (Yang et al., 2025; Kim et al., 2019; Niu et al., 2022). These observations suggest that VDAC plays a regulatory role in DQ-induced ferroptosis, although the precise mechanisms require further investigation.

In conclusion, DQ induces lipid peroxidation and initiates an inflammatory cascade through its redox reactions, culminating in mitochondrial dysfunction and the onset of ferroptosis. Ferroptosis, a pivotal mechanism underlying diquat toxicity, is concomitant with an increase in oxidative stress, thereby exacerbating mitochondrial damage. Additionally, iron overload exacerbates oxidative stress, further aggravating mitochondrial dysfunction. Targeting the ferroptosis pathway has emerged as a promising therapeutic approach for diquat poisoning and may provide novel strategies for mitigating its clinical consequences, particularly in relation to renal or other organ damage.

### 5.2 DQ triggered abnormalities in substance and energy metabolism

Beyond triggering regulated cell death, DQ-induced oxidative stress profoundly disrupts major metabolic pathways, including lipid, amino acid, protein, nucleic acid, and energy metabolism. The overaccumulation of ROS not only compromises mitochondrial integrity and function but also triggers systemic oxidative stress, causing extensive damage to cellular components, including lipids, proteins, nucleic acids, and carbohydrates.

#### 5.2.1 DQ triggered lipid peroxidation *via* dysregulation in ROS

One major consequence of DQ-induced oxidative stress is abnormal lipid peroxidation. Mitochondria play a crucial role in fatty acid β-oxidation and are also a major source of ROS. Under normal physiological conditions, mitochondria efficiently generate ATP through oxidative phosphorylation (OXPHOS), maintaining a balanced production of ROS. However, during metabolic stress, especially when the liver is exposed to an overload of lipids, this balance is disturbed. Upon exposure to DQ, the mitochondrial electron transport chain (ETC.) undergoes adaptive alterations, which include downregulation of upstream components like cytochrome c oxidase, while fatty acid oxidation capacity is either preserved or enhanced (Shi et al., 2024). These changes increase electron leakage in the, ETC., resulting in excessive ROS production, including O_2_•^-^ and H_2_O_2_ (Su et al., 2019). Fatty acid β-oxidation is a major source of ROS, particularly in hepatocytes, when excess free fatty acids (FFAs) enter the mitochondria (Lu et al., 2024). When the oxidative capacity of the mitochondria is overwhelmed by high FFAs, these fatty acids are redirected to peroxisomes and the endoplasmic reticulum. Enzymes like xanthine oxidase (XO) and NADPH oxidase (NOX) in these organelles also generate significant amounts of ROS (Lu et al., 2024). ROS are not only catalysts for lipid peroxidation (LPO), but they also participate in the initiation and propagation of the LPO chain reaction. Hydroxyl radicals (L·) are formed from polyunsaturated fatty acids (LH), which then react with O_2_ to produce lipid peroxides (LOOH) (Valavanidis et al., 2006). Excessive ROS generation due to DQ exposure disrupts cellular radical scavenging mechanisms, initiating the LPO reaction that increases membrane permeability and heightens cellular vulnerability (Osburn et al., 2006). Malondialdehyde (MDA) is a widely recognized biomarker for LPO (Wu et al., 2019; Jia et al., 2020). Previous studies show that MDA levels rise significantly after DQ exposure, and the resulting peroxides can inactivate membrane-bound receptors or alter enzyme structures (Wu et al., 2019; Wang J. et al., 2019). Furthermore, research has revealed that upregulation of the mitochondrial 3-hydroxy-3-methylglutaryl-CoA synthase (HMGCS2) gene counteracts fatty acid oxidation (Sikder et al., 2018). This gene’s upregulation has been observed in the livers of pigs exposed to DQ, indicating that the body activates antioxidant mechanisms in response to lipid peroxidation damage (Wang J. et al., 2019). Although these mechanisms are limited, they reflect an adaptive effort to mitigate the damage caused by lipid peroxidation.

Lipid peroxidation remains a central characteristic of DQ-induced toxicity and target organ damage. Future research may explore its potential as a therapeutic strategy to alleviate DQ-induced organ injuries. Studies have demonstrated that DQ exacerbates oxidative stress and lipid peroxidation both *in vitro* and *in vivo*, accelerating iron accumulation (Cheng et al., 2024). Treatment with deferoxamine (DFO) has been shown to reverse this process and improve spermatogenesis in DQ-poisoned mice (Cheng et al., 2024). DQ-induced hepatic oxidative damage is reflected in elevated ALT and AST levels, enhanced lipid metabolism, and reduced liver fibrosis (Huang et al., 2025). Additionally, DQ induces ROS and lipid peroxidation in primary cultured hippocampal neurons, leading to neuronal injury (Huang et al., 2025). In contrast, Isobenzofuran-1 (3H)-one derivative (compound 1) increases GSH levels in the brains of DQ-treated rats, reducing lipid peroxidation and carbonylated protein levels, thereby offering neuroprotective effects against oxidative damage (Ribeiro et al., 2025). Current research on targeting lipid peroxidation to mitigate DQ-induced renal injury is limited. However, existing studies suggest that the enzyme acid sphingomyelinase-like phosphodiesterase 3b (SMPDL3b) can reduce O_2_•^-^ production and NOX activity, alleviating ROS-induced lipid peroxidation in radiation-induced podocyte damage (Geng, 2021). Furthermore, the COX-2 inhibitor meloxicam has been shown to prevent DQ-induced apoptosis and mitochondrial dysfunction (Choi et al., 2018). These findings highlight potential therapeutic avenues for treating DQ-induced renal and other target organ injuries.

#### 5.2.2 DQ-induced disruption of amino acid and protein metabolism

DQ exposure induces pronounced mitochondrial dysfunction, which in turn disrupts cellular redox homeostasis and promotes the excessive generation of ROS and RNS. The accumulation of ROS and RNS facilitates protein misfolding and oxidative modifications, ultimately compromising protein structure and function (Valavanidis et al., 2006). Kelemen et al. reported that DQ treatment reduced mitotic activity and caused early-stage mitotic arrest in PP2A mutant cells, implicating oxidative stress–mediated perturbations of cell-cycle progression (Ribeiro et al., 2025). Consistently, in *C. elegans*, Wang, B. et al. observed a marked downregulation of mitochondrial complex II and an upregulation of complex III under high DQ exposure, suggesting that alterations in complexes I and III are critical contributors to mitochondria-derived ROS generation (Wang B. et al., 2025). Several studies indicate that activation of antioxidant pathways may attenuate DQ-induced toxicity. For example, quercetin was shown to mitigate DQ-induced cellular injury by enhancing Nrf2 abundance and promoting GSH-dependent redox homeostasis (Jia et al., 2021). Similarly, Cao et al. demonstrated that DQ significantly reduced the activities of mitochondrial complexes I–IV in the jejunum and suppressed the expression of the mitochondrial quality control proteins PINK1 and Parkin, whereas resveratrol effectively reversed these effects (Cao et al., 2019). These findings underscore the importance of antioxidant defense activation in counteracting DQ-induced oxidative damage. Excessive ROS not only impairs protein synthesis but also reduces ATP production, thereby altering the expression and enzymatic activity of multiple intracellular proteins. Indeed, DQ exposure has been shown to significantly modulate the levels and activities of SIRT1, Bcl-2, and mitochondrial complexes I–IV (Bhattacharyya et al., 2014). Amino acid residues are also vulnerable to ROS-mediated oxidation, with methionine being particularly susceptible. Methionine readily reacts with ROS to form methionine sulfoxide, functioning as an intrinsic ROS scavenger. Elevated methionine sulfoxide levels and disrupted methionine metabolism further indicate substantial oxidative stress in DQ-exposed organisms (Hu and Du, 2023).Collectively, these findings highlight that DQ-induced ROS overproduction broadly disrupts intracellular protein function. Since these proteins play pivotal roles in essential physiological and pathological processes, including autophagy, necroptosis, and ferroptosis—their dysregulation may profoundly compromise cellular homeostasis.

#### 5.2.3 DQ-induced disruption of nucleic acid metabolism

DQ significantly impacts nucleic acid metabolism by generating ROS, which lead to DNA damage, chromosomal abnormalities, and disruptions in the cell cycle (Valavanidis et al., 2006). These reactive species not only directly damage DNA but also have the potential to induce gene mutations and impair cellular function. In addition to promoting DNA damage, DQ suppresses the expression of key regulatory factors involved in antioxidant defense. Specifically, DQ downregulates the mRNA levels of Nrf2 and its target genes in various organs, including the liver, kidneys, and intestines (Qi et al., 2018; Silva-Palacios et al., 2025; Wu F. et al., 2024). Nrf2 is a pivotal transcription factor that governs the expression of antioxidant enzymes, and its decreased expression may compromise the cellular antioxidant defense system, thereby exacerbating oxidative stress (Motohashi and Yamamoto, 2004). Furthermore, DQ exposure leads to a significant reduction in the mRNA levels of several antioxidant enzymes, including NAD(P)H:quinone oxidoreductase 1 (NQO1), SOD1, glutathione S-transferase α1 (GSTA1), heme oxygenase-1 (HO-1), GPX1, and thioredoxin reductase 1 (TXNRD1) (Lightfoot et al., 2006). These enzymes are crucial for the scavenging of free radicals and the repair of oxidative damage. The downregulation of these enzymes by DQ suggests that oxidative stress-induced alterations in gene expression may disrupt nucleic acid metabolism, thereby contributing to cellular dysfunction (Lightfoot et al., 2006).

Moreover, DQ has been shown to induce differential expression of long non-coding RNAs (lncRNAs) and mRNAs in pig liver, with notable changes in genes involved in oxidative stress and immune responses (Wang J. et al., 2019). Specifically, the immune-related gene ILF3 is upregulated following DQ exposure (Wang J. et al., 2019). Further analysis revealed that these gene expression changes are associated with the modulation of at least 10 signaling pathways, with the AMP-activated protein kinase (AMPK) pathway being particularly prominent (Wang J. et al., 2019). The AMPK pathway is integral to cellular energy regulation and antioxidant responses (Hsu et al., 2022), and its dysregulation may influence cellular metabolic state and the ability to cope with oxidative stress (He et al., 2018; Lin et al., 2025).

In conclusion, DQ induces the generation of ROS, inhibits antioxidant stress responses, and alters the expression of genes involved in nucleic acid metabolism, cell cycle regulation, and immune function. Continued research into the molecular mechanisms underlying DQ-induced toxicity, particularly its effects on nucleic acid metabolism, could provide valuable insights for developing targeted intervention strategies to mitigate its harmful effects.

#### 5.2.4 DQ-induced disruption of energy metabolism

DQ has a profound and multifaceted impact on the body’s energy metabolism. A study by Hu et al., employing LC-MS analysis, demonstrated that DQ intoxication significantly altered the levels of metabolites related to energy metabolism (Hu and Du, 2023). Specifically, the DQ-exposed group showed a decrease in succinate levels and an increase in isocitrate levels (Hu and Du, 2023). Both succinate and isocitrate are critical intermediates in the tricarboxylic acid (TCA) cycle, a central metabolic pathway that primarily occurs on the inner mitochondrial membrane of eukaryotic cells and is essential for cellular energy production (Tsvetkov et al., 2022). Disruption of the TCA cycle not only impairs energy production but also hinders the synthesis of key metabolic intermediates for essential biosynthetic pathways, including those for carbohydrates, lipids, and proteins. Therefore, disturbances in the TCA cycle due to DQ exposure are closely linked to mitochondrial dysfunction and oxidative stress, suggesting that DQ toxicity may impede mitochondrial activity, thereby disrupting the TCA cycle and compromising cellular energy supply (Cao et al., 2019; Aranda-Rivera et al., 2021). In addition to TCA cycle disruption, alterations in galactose metabolism play a critical role in DQ-induced toxicity. Galactose metabolism predominantly occurs in the liver through the Leloir pathway, resulting in the formation of 1-phosphate glucose, which is further processed *via* the pentose phosphate pathway to produce reduced NADPH (Tsevelkhorloo et al., 2021). NADPH is a vital cofactor involved in the biosynthesis of fatty acids, steroid hormones, and amino acids (Coelho et al., 2015). Research has shown that DQ treatment leads to elevated levels of fatty acids, steroid hormones, and their derivatives, suggesting that DQ toxicity consumes NADPH, converting it into DQ^+^ and facilitating the generation of ROS and RNS through redox cycles (Han et al., 2023). This process further exacerbates oxidative stress and cellular damage. Moreover, DQ exposure was found to increase the levels of galactitol and sorbitol, potentially due to an alternative metabolic pathway for galactose, wherein aldose reductase converts galactose into galactitol (Hu and Du, 2023). This suggests that the increase in galactitol may reflect a compensatory metabolic response to DQ toxicity (Hu and Du, 2023). Metabolomic analyses also revealed a significant decrease in the levels of myo-inositol in the DQ-treated group (Hu and Du, 2023). Myo-inositol and its derivatives play key roles in various biological processes, including glucose metabolism, cell signaling, gene transcription, and apoptosis (Dinicola et al., 2021). A deficiency in myo-inositol may trigger apoptotic pathways through the PI3K/Akt signaling cascade and has been implicated in neurodegenerative diseases such as Alzheimer’s and Parkinson’s disease (Xiao et al., 2022; Bizzarri et al., 2016). In conclusion, DQ intoxication induces oxidative stress and disrupts key metabolic pathways, leading to dysfunction in several target organs, including the gastrointestinal tract, nervous system, liver, and kidneys. Targeting the metabolic disturbances caused by DQ toxicity represents a promising strategy for mitigating the associated organ damage.

## 6 Targeted therapeutic approaches for DQ toxicity

Currently, no specific antidote has been identified for DQ poisoning, making the search for effective therapeutic strategies an important area of research. Oxidative stress dysregulation is considered one of the primary mechanisms underlying DQ toxicity and damage to its target organs. Consequently, therapeutic approaches targeting this mechanism have become crucial for alleviating DQ poisoning and improving target organ injury (Table 1). N-acetylcysteine (NAC), a small molecule, exhibits antioxidant properties through its active thiol group (-SH), which scavenges free radicals. Upon entering cells, NAC is deacetylated and converted into a precursor for glutathione synthesis, thereby effectively replenishing the intracellular pool of reduced glutathione, inhibiting oxidative stress, and ameliorating early symptoms of toxicity (Cotgreave et al., 1987; Kwok et al., 2023). Studies have shown that GSH, as the primary intracellular reductant, can significantly mitigate DQ-induced oxidative stress and improve organ damage, including damage to the gastrointestinal tract (Liang et al., 2023). Additionally, resveratrol (RSV), a natural antioxidant, has been shown to reduce the damage to antioxidant enzyme activity induced by DQ, decrease ROS production, alleviate mitochondrial depolarization, improve mitochondrial morphology, and promote mitochondrial mitophagy, thereby providing protection to both the gastrointestinal and nervous systems (Zhang H. et al., 2020; Chen et al., 2021; Yuan et al., 2016). Further research has revealed that RSV derivatives, such as pterostilbene (PT), exhibit superior effects in preventing mitochondrial damage (Yuan et al., 2016). PT enhances the expression and activity of Nrf2 and SOD, activates Sirtuin 1, prevents mitochondrial swelling, membrane potential collapse, and ATP depletion, and suppresses MDA levels, thus demonstrating potent antioxidant capabilities (Zhang H. et al., 2020). Curcumin has been shown to promote Parkin-dependent mitophagy *via* the AMPK-TFEB signaling pathway, improving intestinal barrier damage and mitochondrial dysfunction induced by oxidative stress (Cao et al., 2020). Moreover, selenoprotein V has been identified for its antioxidant function, as it can facilitate the reduction of other proteins through cysteine thiol-disulfide exchange, thus mitigating oxidative damage and endoplasmic reticulum stress induced by DQ (Zhang X. et al., 2020; Chen et al., 2020). In terms of drug selection, other compounds, such as arginine (Arg) and N-carbamoylglutamate (NCG), are considered to alleviate DQ-induced oxidative stress by enhancing antioxidant enzyme activity and upregulating the expression of SOD, GPX1, and Nrf2 (Gonzalez et al., 2023). Furthermore, 0.15% tryptophan (Trp) has been shown to strengthen the integrity of the intestinal barrier in piglets exposed to DQ (Liu et al., 2019), improving intestinal function, redox status, and mitochondrial function, while also modulating protein synthesis and immune responses (Liang et al., 2018; Shen et al., 2012). Functional probiotics, taurine, dietary puerarin, squalene, and other compounds have also been reported as potential antioxidants, with the promise of becoming part of future therapeutic strategies (Wen et al., 2020; Wu et al., 2019; Liu et al., 2020; Kanthasamy et al., 2012). Although studies indicate that these compounds significantly reduce oxidative stress and inflammation in DQ-poisoned animals and improve toxicity symptoms, their effectiveness for clinical treatment still requires further investigation and validation. Therefore, future research should focus on exploring multi-target therapeutic strategies that encompass key biological processes such as oxidative stress, mitochondrial function, cell death, and metabolic pathways, with the aim of providing new theoretical foundations and clinical approaches for the prevention and treatment of DQ toxicity.

**TABLE 1:** Targeted therapeutic approaches for DQ toxicity.

Mechanism	Intervention	Target/signaling pathway	Effect for target/signaling pathway	*In-vivo* model	*In-vivo* subject	*In-vitro* model	*In-vitro* subject	Results	Target organ	Reference
Pyroptosis	Ferulic acid (FA)	Caspase-1	Inhibition	Diquat	Piglets	-	-	Inhibition	Liver	Luo et al. (2024)
​	Deferoxamine	gasdermin E	Inhibition	-	-	Diquat	Human renal tubular epithelial cells (HK-2 cells)	Inhibition	Kidney	Chen et al. (2024)
​	selenium nanoparticles (SeNPs)	AMPK/NLRP3/Nrf2	inhibition	Diquat	Mouse and Piglet models	-	-	inhibition	intestinal	Qiao et al. (2025)
​	Epigallocatechin gallate (EGCG)	NF-κB/NLRP3	inhibition	-	-	Diquat	HK-2 cells	inhibition	Kidney	Qu et al. (2024)
Autophagy	pifithrin-α (PFT-α)	LC3-II, caspase-3,p53	inhibition	-	-	Diquat	PC12 cells	Upregulation	Nervous system	Park and Koh (2019)
​	Curcumin	AMPK-TFEB	upregulation	Diquat	Piglet	Diquat	porcine intestinal epithelial cells (IPEC-J2 cells)	Upregulation	Intestinal	Cao et al. (2020)
​	Hydroxytyrosol (HT)	PI3K/Akt-Nrf2	upregulation	Diquat	Piglet	Diquat	IPEC-J2 cells	Upregulation	intestinal	Wen et al. (2024)
​	BAI-nanoliposome (BAI-NL)	putative kinase 1 (PINK1)/Parkin	upregulation	Diquat	Mouse	Diquat	AML12 hepatocytes	Upregulation	liver	Zhou et al. (2025)
​	*Bacillus* amyloliquefaciens SC06 (BaSC06)	AKT/FOXO	inhibition	-	-	Diquat	IPEC-J2 cells	Upregulation	intestinal	Tang et al. (2021)
​	Probiotics	p38 MAPK	upregulation	Diquat	Sprague-Dawley (SD)	-	-	Upregulation	intestinal	Wu et al. (2019)
​	Caulerpa lentillifera (CLE)	Nrf2	Upregulation	Diquat	Zebrafish	-	-	Upregulation	liver	Lin et al. (2025)
​	Resveratrol(RSV)	PINK1/parkin	upregulation	Diquat	Piglets	-	-	Upregulation	intestinal	Cao et al. (2019)
​	AMG487	CXC-motif chemokine receptor 3 (CXCR3)	Inhibition	diquat	Mouse	Diquat	IPEC-J2 cells	Upregulation	intestinal	Gao et al. (2025)
​	Dietary Tributyrin	PINK1/Parkin	Upregulation	Diquat	Piglets	-	-	Upregulation	intestinal	Wang C. et al. (2019)
Ferroptosis	glycine	SLC7A11	Inhibition	Diquat	Piglets	-	-	Inhibition	Liver	Hua et al. (2022)
​	Edaravone (Eda)	Aldh1l1 m6A methylation	Upregulation	Diquat	Mouse	Diquat	PC12	Inhibition	Nervous system	Wu et al. (2025)
​	Pterostilbene	Nrf2	Upregulation	Diquat	Broiler chickens	-	-	Inhibition	Intestinal	Chen et al. (2023)
​	DFO	GPX4,SLC7A11	Upregulation	Diquat	Mouse	Diquat	HK-2	Inhibition	Kidney	Sun et al. (2025)
​	Holly (Ilex latifolia Thunb.) polyphenols extracts (HPE)	GPX4,SLC7A11	Upregulation	Diquat	Piglets	-	-	Inhibition	Liver	He et al. (2020)
​	Polyphenols sourced from Ilex latifolia Thunb. (PIT)	GPX4,SLC7A11,GSH	Upregulation	Diquat	Piglets	-	-	Inhibition	Intestinal	Xu et al. (2022)
​	Lactiplantibacillus plantarum	PI3K/AKT NF-κB	Upregulation	Diquat	Piglets	-	-	Inhibition	Intestinal	Liu et al. (2024)
​	Fer-1	VDAC1, FTMT	Inhibition	Diquat	Zebrafish	-	-	Inhibition	kidney	Ou et al. (2025)
​	deferoxamine (DFO)	HO-1	Inhibition	Diquat	Mouse	-	-	Inhibition	Reproductive system	Cheng et al. (2024)
Lipid Metabolism	selenium	Lipid peroxidation	Inhibition	Diquat	Rat	-	-	Inhibition	liver	Burk et al. (1980)
​	Melatonin	Lipid peroxidation	Inhibition	Diquat	Rat	-	-	Inhibition	liver	Zhang et al. (2006)
​	L-NAME	Lipid peroxidation	Inhibition	Diquat	Wistar rat	-	-	Inhibition	Nervous system	Djukic et al. (2012)
​	RSV	Lipid peroxidation	Inhibition	Diquat	Piglets	-	-	Inhibition	Liver	Zhang H. et al. (2020)
​	pterostilbene (PT)	Lipid peroxidation	Inhibition	Diquat	Piglets	-	-	Inhibition	Liver	Zhang H. et al. (2020)
​	isobenzofuran-1(3H)-one	Lipid peroxidation	Inhibition	Diquat	Wistar rats	Diquat	Hippocampal neurons	Inhibition	Nervous system	Ribeiro et al. (2025)
​	N-Carbamylglutamate (NCG)	Lipid peroxidation	Inhibition	Diquat	Rats	-	-	Inhibition	Liver	Cao et al. (2016)

Summary of potential therapeutic agents for Diquat (DQ) toxicity, their proposed mechanisms of action, and key protective effects in DQ models (with corresponding references).

## 7 Future Perspectives

Despite significant progress in understanding DQ toxicity, several critical knowledge gaps remain and warrant further investigation. First, the precise molecular mechanisms linking DQ-induced oxidative stress to specific regulated cell death pathways, particularly pyroptosis, ferroptosis, and mitophagy, required deeper exploration. While evidence supports the activation of NLRP3 inflammasome and gasdermin-mediated pyroptosis in DQ models, the upstream signaling events (e.g., whether TXNIP/TRX dissociation directly contributes to NLRP3 activation in DQ toxicity) have not been conclusively demonstrated. Future studies using genetic knockout models and specific inhibitors are needed to dissect these pathways.Second, the role of mitochondrial dysfunction as a driver rather than a consequence of DQ-induced ROS production should be further clarified. Advanced techniques such as real-time mitochondrial membrane potential monitoring, high-resolution respirometry, and mitochondrial-targeted antioxidants may help establish causality.Third, multi-omics approaches (transcriptomics, proteomics, metabolomics, and lipidomics) applied to DQ-exposed animal models and patient samples hold promise for identifying novel biomarkers of exposure, early organ injury, and prognosis. Such integrated analyses could reveal previously unrecognized pathways and therapeutic targets.Fourth, the translational potential of currently identified antioxidants (e.g., resveratrol, curcumin, N-acetylcysteine) and mitophagy-modulating agents (e.g., butyrate) should be rigorously evaluated in large-animal models and, eventually, clinical trials. Combination therapies targeting multiple nodes of the oxidative stress–cell death–metabolism network may offer superior efficacy. Fifth, the long-term health consequences of subacute or chronic DQ exposure, particularly in agricultural workers and populations living near treated fields, remain understudied. Epidemiological studies are urgently needed to assess the risk of neurodegenerative diseases, chronic kidney disease, and intestinal disorders associated with low-level DQ exposure.Addressing these knowledge gaps will not only advance our fundamental understanding of DQ toxicology but also pave the way for effective preventive and therapeutic strategies against DQ poisoning.

## 8 Conclusion

In summary, oxidative stress dysregulation plays a central role in the occurrence and progression of DQ toxicity and its associated damage to target organs. Among the mechanisms involved, regulated cell death and disruptions in substance and energy metabolism are crucial, with mitochondrial dysfunction being one of the core pathological mechanisms directly affecting the survival and function of cardiomyocytes. The oxidative stress dysregulation in DQ toxicity and target organ damage also interferes with normal mitochondrial function through multiple pathways, manifesting as regulated cell death (including pyroptosis, autophagy, and ferroptosis) as well as abnormalities in energy and substance metabolism. Firstly, DQ-induced oxidative stress dysregulation triggers pathological changes such as a decrease in mitochondrial membrane potential, impaired ATP synthesis, and increased ROS production. These changes directly lead to mitochondrial dysfunction, thereby initiating regulated cell death. Secondly, excessive ROS, as a stress factor, induces lipid peroxidation, abnormal protein synthesis and activity, and disruptions in energy metabolism, which further aggravates target organ damage. Currently, several biomolecules and phytochemicals have shown protective effects against DQ-induced toxicity in experimental models; however, their clinical efficacy and safety remain to be validated. Key unresolved questions and future research priorities are presented in the Future Perspectives section.
